# Use of a smartphone app combined with gamification to increase the level of physical activity of adults and older adults: protocol of a sequential multiple assignment randomized trial

**DOI:** 10.1186/s13063-019-3879-1

**Published:** 2019-12-27

**Authors:** Maria do Socorro Morais Pereira Simões, Bárbara de Barros Gonze, Neli Leite Proença, Vinicius Tonon Lauria, Vinícius Demarchi Silva Terra, Ricardo da Costa Padovani, Victor Zuniga Dourado

**Affiliations:** 1 Departamento de Ciências do Movimento Humano, Universidade Federal de São Paulo, Instituto de Saúde e Sociedade, 136 Silva Jardim st – room 338, Santos, SP 11015-020 Brazil; 2 Departamento de Saúde, Educação e Sociedade, Universidade Federal de São Paulo, Instituto de Saúde e Sociedade, 136 Silva Jardim st – room 215, Santos, SP 11015-020 Brazil

**Keywords:** Cardiovascular diseases, physical activity, smartphone

## Abstract

**Background:**

There is scientific evidence suggesting that app-based interventions targeted to increase the level of physical activity might be effective, although multicomponent interventions appear to be more effective than app-based interventions alone. Despite the motivating results, it remains unclear whether or not app-based interventions can increase the level of physical activity and cardiovascular health. Our study aims to investigate the effect of a smartphone app combined with gamification on the level of physical activity of adults and older adults. The specific aims are (1) to verify the effects of the intervention on cardiometabolic and cardiovascular health, lung function, and cardiorespiratory fitness; and (2) to verify the relationship between age group and the response rate.

**Methods/design:**

We will conduct a sequential multiple assignment randomized trial (SMART). The adaptive intervention protocol will last 6 months. After baseline assessments, participants will be randomized into one of three groups (group 1: app + tailored messages; group 2: app + tailored messages + gamification I; control group: physical activity counseling). For 12 weeks, we will record the average number of steps per day of participants from groups 1 and 2. At 6 weeks from initiation of recording, participants will be classified into responders and non-responders according to their increase in the average number of daily steps; all those considered as non-responders will be re-randomized, with the chance to participate in a third group – group 3: app + tailored messages + gamification II. Finally, at 12 weeks, participants will continue using the app but will no longer receive direct intervention from investigators. All participants will be reassessed at 3 and 6 months from baseline. Our pilot SMART will require 42 participants (14 per arm). Following the SMART pilot, we will calculate the sample size for the trial based on the variation of the average number of steps/day, including an up to 40% loss to follow-up and a less optimistic nonresponse rate of 65%.

**Discussion:**

To our knowledge, this will be the first trial with adaptive intervention to test the effectiveness of using a smartphone app to increase the level of physical activity of adults and older adults.

**Trial registration:**

Brazilian Clinical Trials Registry: RBR-8xtc9c. Registered on 3 August 2018, http://www.ensaiosclinicos.gov.br; UTN number: U1111–1218-1092.

## Background

Popular technologies such as smartphones and their applications (apps) have been used as tools to engage people who do not meet the recommended levels of physical activity. There is scientific evidence with moderate effect (weighted effect size = 0.54) suggesting that app-based interventions targeted to increase the level of physical activity might be effective [1], although multi-component interventions appear to be more effective than app-based interventions alone [2]. Apps intended to improve cardiovascular health should include features such as privacy policy and reliable sources of information and should be mainly designed using behavior change theory [3]. It is believed that the development of apps based on behavioral change techniques may contribute to the emergence of cognitive patterns favorable to the practice of physical activity.

The main effective components of apps are most likely personalization and gamification [4]. Interestingly, the adherence rate to an app-based intervention was not significantly different in comparison to conventional intervention [5]. However, despite its increasing use, smartphone apps for physical activity lack a scientific basis. Among many health and fitness apps, only a few are based on behavior change techniques [2, 6–8].

The use of games for promoting healthy lifestyle behaviors (such as physical activity) has been shown to be effective [9], introducing gaming elements as novel opportunities for behavior change techniques, increasing enjoyment and, thus, intrinsic motivation. The use of game elements outside of the game context is called gamification and has been increasingly used for promoting healthy lifestyle behaviors. Among gaming elements, goal-setting with feedback and rewards are the most frequently used. Social connection, progress bars, and challenges/quests have also been investigated in a smaller number of studies [10]. Studies have also shown that there is a limited theoretical foundation for gamification, and most studies have used goal-setting as a motivation strategy to engage people in playing the game [10].

Among the main limitations of the available apps, with or without game elements, is the long-term adherence to app-based interventions [10, 11]. Despite the motivating results, it remains unclear whether or not app-based interventions can increase the level of physical activity and cardiovascular health.

## Methods

This study protocol was written according to the Standard Protocol Items: Recommendations for Interventional Trials (SPIRIT) 2013, and the SPIRIT checklist is available in Additional file 1.

### Aims and design

The primary aim of our study is to investigate the effect of a smartphone app combined with game elements on the level of physical activity of adults and older adults, measured by the average number of daily steps. The secondary aims are (1) to verify the effects of the intervention on cardiometabolic and cardiovascular health, lung function, and cardiorespiratory fitness; and (2) to verify the relationship between age group and the response rate.

We will conduct a Sequential Multiple Assignment Randomized Trial (SMART). This study was approved by the Ethics Committee of the Federal University of Sao Paulo (# 0499/2018) and is registered in the Brazilian Clinical Trials Registry (RBR-8xtc9c).

The SMART design has been recommended over the classical randomized controlled trial for technology-based interventions [12, 13] as it allows adaptations over time based on the participants’ response to the intervention. Despite the large potential and shown feasibility [14], the SMART design has, thus far, hardly been conducted in smartphone and gamified interventions.

**Fig. 1:**
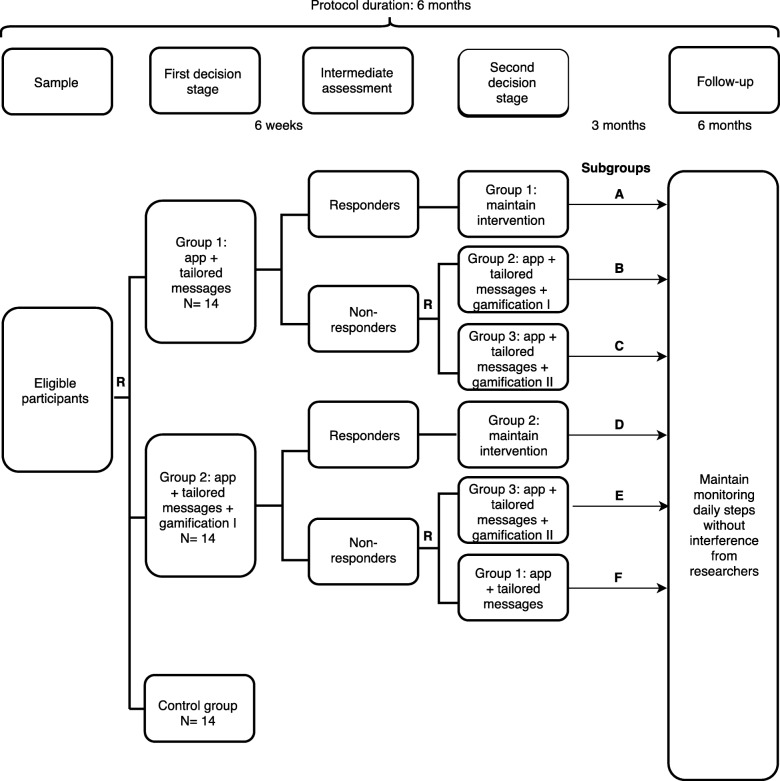
Study flowchart

**Fig. 2:**
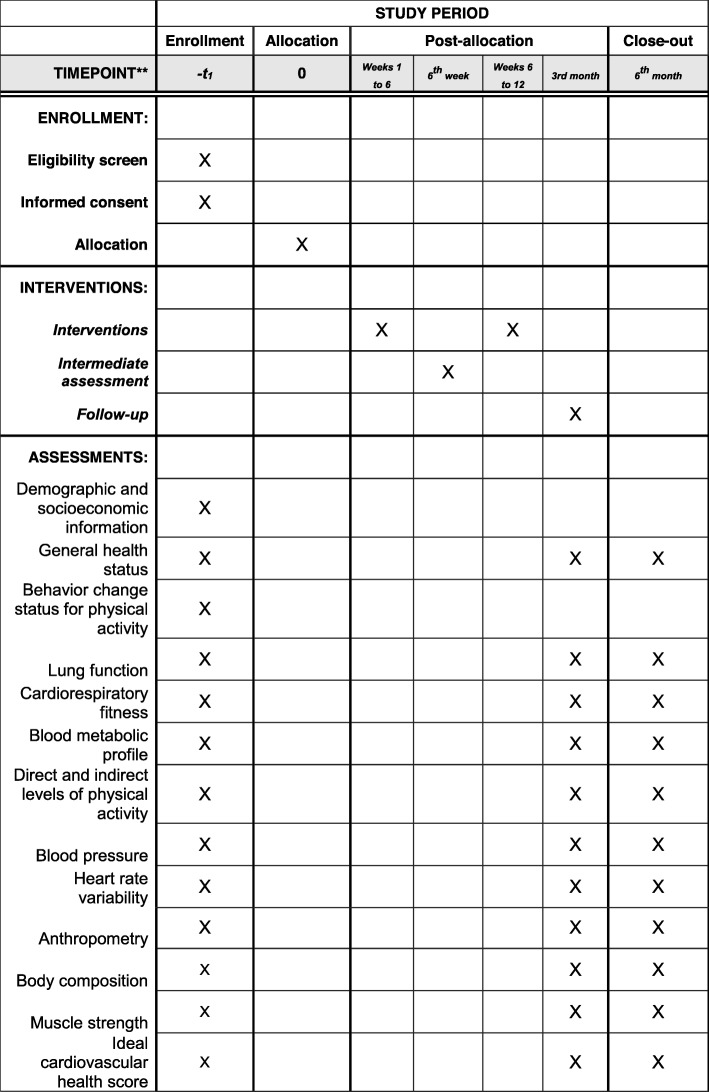
SPIRIT figure – protocol timeline

The adaptive intervention protocol will last 6 months. Initially, participants will be randomized into one of three groups (group 1: app + tailored messages; group 2: app + tailored messages + gamification I; control group: physical activity counseling) (Fig. 1). Over 12 weeks, we will record the average number of steps per day for each participant from groups 1 and 2. At 6 weeks from initiation, participants will be classified into responders and non-responders according to the targeted average number of daily steps; all those considered as non-responders will be re-randomized, with the chance to participate in a third group – group 3: app + tailored messages + gamification II (Fig. 1). At the end of the 12 weeks, participants will continue using the app but will no longer receive any direct intervention from investigators. All participants, including those in the control group, will be reassessed at 3 and 6 months from baseline. The protocol timeline is shown in Fig. 2.

Assessors will be blinded to group allocation. We will randomize participants in blocks of six (two to each group) using opaque sealed envelopes and the sequence of group allocation will be computer generated.

Before randomization, all participants will watch a brief animated video about the benefits of practicing physical activity, the minimum quantity of exercise recommended to reach health benefits, and examples of daily-living situations where they can increase their levels of physical activity. They will receive a link to watch the video on the internet as many times as they want and will receive a printed booklet with the same content of the video.

Participants from groups 1 and 2 will have a commercial freely available app installed on their smartphones. This app has gamification functionalities, and it will track their daily steps. Participants will be instructed about the use and functionalities of the app, and they will be encouraged to use the smartphone close to their waist during all activities.

### Interventions

#### Group 1 – App + tailored messages

Participants will use the app to track the number of daily steps and they will receive weekly text messages on their smartphones, with information about their performance on last week and motivational phrases according to their behavior-change status at the end of the protocol, the responders of Group 1 will end up in the subgroup A, while non-responders will end up in the subgroups B or C.

#### Group 2 – App + tailored messages + gamification I

Additionally to monitoring the number of steps and individual messages, participants will be encouraged to join virtual challenges; for each challenge they complete, a virtual badge will be given. The challenges available will be related to the target number of steps per day, target distance walked in the month, and group competitions where the total number of steps is compared among the different groups of app users. Further, there will be rankings of the number of steps among all users at the end of the protocol, the responders of Group 2 will end up in the subgroup D, while non-responders will end up in the subgroups E or F.

#### Control group

The control group will receive physical activity counseling, provided by reinforcing the information of the video they watched prior to randomization.

#### Group 3 – App + tailored messages + gamification II

The second stage of gamification will be available in the protocol by the time of the intermediate assessment (6th week of intervention). Gamification II will include the same features of gamification I, plus opportunities for socialization among participants (biweekly group meetings) and discussion forums about physical activity.

### Defining responders and non-responders

The American College of Sports Medicine recommendations about the quantity and intensity of physical activity suggest increases of 2000 steps per day to achieve improvements in cardiovascular health [15]. After a pre-test with persons from researchers’ networks using a smartphone app with a pedometer, we observed that the target of increasing 2000 steps/day would not be realistic to our population. Therefore, we decided to plot the average number of steps of participants in a graph using a linear tendency line and participants that present a positive slope, i.e., a tendency to increase step counts, will be considered as responders. Consequently, participants with a zero or negative slope, i.e., a tendency to maintain or decrease step counts, will be considered as non-responders. Additionally, we will use the app’s first-week step count as a reference for both sending the tailored messages and analyzing participants’ slopes. Thus, we believe that this way we will be able to set individualized and feasible goals for our population.

#### Intermediate assessment

The intermediate assessment to identify responders and non-responders will take place at week 6 from the beginning of the protocol. We will calculate the average number of steps/day from weeks 1 to 6, and compare with the number of steps/day from the initial assessment. Participants identified as non-responders will then be re-randomized and will receive instructions about the new phase of intervention (Fig. 1). We will collect the average number of steps per day using the app interface; all participants will use the app for 1 week after randomization prior to any intervention and we will consider the step count from the first week as a comparator for weekly and intermediate assessments.

### Follow-up

After 3 months of intervention, all participants (including those in the control group) will be reassessed. Participants from groups 1, 2, and 3 will be advised to maintain the monitoring of daily steps by the app but will no longer receive any direct intervention from researchers (text messages, virtual challenges, or group meetings). Finally, after 3 months of self-monitoring of the number of steps, all participants will be reassessed and the protocol completed.

### Sample size

The main objective of a SMART study relies on the feasibility of carrying out a future large-scale trial, rather than investigating the effects of the proposed set of treatments. To reach this goal, we must ensure that the number of subjects will be sufficient and balanced among all of the subgroups. According to Almirall et al. [16], a fixed probability *k* of about 80–90% should be considered so that at least *m* participants would end in non-responders’ subgroups B, C, E and F (Fig. 1). Considering a range of non-response rates in SMART studies of 35–65%, we calculated an *m* of 3 participants per subgroup as sufficient to ensure familiarity with the protocol, treatment delivery, and identification of potential problems. For a non-response rate of q = 50% at the end of the 6th week, our pilot SMART study will require 42 participants (14 per arm) [16].

After conducting this SMART pilot, we will consider the variation on the average number of steps per day to calculate the sample size of the trial, including an up to 40% loss to follow-up and a less optimistic non-response rate of 65%. To increase the feasibility of a future large-scale trial, we will calculate the sample size considering groups A + B (maintain – switch to group 2), groups A + C (maintain – switch to group 3), groups D + E (maintain – switch to group 1), groups D + F (maintain – switch to group 3), and the control group.

### Population and recruitment

Eligible participants will be community-dwelling adults aged 20 years and older, recruited from social networks, folder distribution, and digital media. The inclusion criteria are age 20 years and older, without previously diagnosed or self-reported cardiopulmonary diseases, locomotor disturbances, or other health problems that would preclude the safe performance of physical activity (e.g., electrocardiographic abnormalities during exercise test), having a smartphone, and being familiar with its use.

The exclusion criteria are average baseline level of physical activity ≥10,000 steps per day, unable to walk without the use of gait-assistive devices, recent respiratory tract infections, spirometric abnormalities, stable or unstable angina in the 4 weeks prior to examination, recent myocardial infarction, previous angioplasty or heart surgery, bradycardia or tachycardia, and refusal to participate. We set the limit of 10,000 steps per day because this is a well-known threshold for improving health [17].

### Study measurements

Participants will be assessed at baseline, and after 3 and 6 months; all assessors will be blinded to group allocation. Each assessment will include demographic and socioeconomic information, general health status, lung function, cardiorespiratory fitness, blood metabolic profile, direct and indirect levels of physical activity, blood pressure, heart rate variability, anthropometry, body composition, ideal cardiovascular health score, and muscle strength.

Assessments will be carried out in 2 days, with a 7-day interval between them. On the first day, we will assess demographic and socioeconomic information, behavior-change status for practicing physical activity, general health status, lung function, and cardiorespiratory fitness. At this point, participants will receive a blood exam form and an accelerometer, which must be used for 7 days. In the second day of assessments, participants will give the accelerometer back and will be given an assessment of their indirect level of physical activity, body composition, heart rate variability, blood pressure, anthropometry, muscle strength, and ideal cardiovascular health score.

#### Assessments

All interviews and tests will be carried out by trained staff and all equipment will be periodically checked regarding calibration.

##### Demographic and socioeconomic information

Age, sex, marital status, completed years of study, and socioeconomic classification according to a Brazilian valid questionnaire [18] will be recorded.

##### General health status

We will record information on personal and family diagnoses, medication use, and cardiovascular risk factors.

##### Behavior-change status for physical activity

To evaluate the status of behavior change we will use an adapted and validated questionnaire [19]. This questionnaire assesses the current physical activity status (practicing or not practicing) and if the person is willing to change it.

##### Lung function

Participants will perform spirometry (Quark PFT/CPET Cosmed, Pavona di Albano, Italy) following international standards [20]. We will register the forced expiratory volume in the first second (FEV_1_), forced vital capacity (FVC), and relation between the two (FEV_1_/FVC).

##### Cardiorespiratory fitness

Participants will perform a cardiopulmonary exercise testing on a treadmill (ATL, Inbrasport, Porto Alegre, Brazil) following a ramp protocol [21]. We will measure ventilatory, cardiovascular and metabolic responses, breath by breath, using a gas analyzer (Quark PFT, COSMED, Italy).

##### Blood metabolic profile

Participants will have collected a blood sample at a certified laboratory. We will register the levels of total cholesterol, HDL-cholesterol, LDL-cholesterol, fasting glucose, and triglycerides.

##### Direct and indirect levels of physical activity

The direct level of physical activity will be assessed by a triaxial accelerometer (Actigraph, Pensacola, USA), which must be used for 7 consecutive days on participants’ dominant hip. We will consider as valid data those registers of at least 4 days including at least 1 day of the weekend, for 10 h per day [22]. We will collect the number of steps per day, time in sedentary behavior, time in light activities, and time in moderate-to-vigorous activities. The accelerometer step count will be used to the exclusion criteria of ≥10,000 steps/day. The indirect level of physical activity will be calculated using the International Questionnaire of Physical Activity (IPAQ) in its long form [23].

##### Blood pressure

Participants will be comfortably sat and we will register three blood pressure measurements with a 1-min interval between them.

##### Heart rate variability

Participants will be resting in a supine position. We will register the heart rate variability for 10 min using a cardiac monitor (RS800, Polar Electro, Kempele, OY, Finland). The interval between R-waves will be analyzed using the software Polar ProTrainer 5 (Polar Electro™, OY, Kempele, Finland).

##### Anthropometry

We will measure participant height (m), body mass (kg), and abdominal circumference (cm). We will also calculate the body mass index in kg/m^2^.

##### Body composition

To assess body composition, we will use a bioelectrical impedance analyzer (310e Biodynamics, Detroit, USA), registering resistance, reactance, lean body mass (kg), and the percentage of body fat.

##### Muscle strength

We will measure muscle strength by the handgrip strength using a hydraulic hand dynamometer (Saehan, Japan), with the participant in a seated position [24]. We will register the highest value of the three measurements.

##### Ideal cardiovascular health score

The ideal cardiovascular health score will be calculated according to the American Heart Association recommendations [25].

### Data analysis

Participants’ characteristics will be presented by descriptive statistics. We will use ANOVA tests to compare the effect of interventions on the increase of physical activity levels, cardiovascular and metabolic health, lung function, and cardiorespiratory fitness among groups A + B + C and D + E + F pre- and post-intervention compared to the control group. Additionally, we will use the same test to analyze the effect of interventions on the average number of daily steps comparing the final subgroups (A to F) against the control group as well as the subgroups A + F, B + D and C + E against the control group.

To analyze the relationship between age and the response rate to each intervention, we will group responders and non-responders according to age ranges (20–30 years old, 31–40, 41–50, 51–60, 61–70, and 71 and older) and compare them in subgroups from A to F using Pearson or Spearman correlations (according to data distribution).

## Discussion

To our knowledge, this will be the first trial with an adaptive intervention to test the effectiveness of using a smartphone app to increase the level of physical activity of adults and older adults. As stated above, the SMART design has been recommended over the classical randomized controlled trial for technology-based interventions [12, 13].

One of the major challenges of the present study will be to engage Brazilians to track their daily steps using technology. Despite the large number of smartphones in use in Brazil, whether the proposed application will be effective in this population needs more clarification. Another challenge will be the lack of security to carry out outdoors physical activities in Brazil, regardless of the neighborhood. In our recent experience, many participants from our pre-test reported not using a smartphone when practicing physical activity outdoors. We believe that creating favorable conditions for behavior change and the adoption of a healthy lifestyle will favor adherence and engagement of those involved in the study. Using the technology available on a smartphone to encourage the adoption of new ways of thinking and acting can be a new way of promoting the quality of life and well-being of the Brazilian population.

Finally, the sample size required for SMART studies is usually large and difficult to calculate. We are aware of the difficulty of involving enough people in a tight timeline. However, we are confident that we are prepared to run the protocol with sufficient statistical power, at least for some key health variables.

### Trial status

The study is currently recruiting participants. Recruitment began on 15 October 2018, and is intended to be completed on 15 November 2020.

**Protocol version and date:** Version number 4, 23 October 2019.

## Supplementary information

**Additional file 1.** SPIRIT checklist.

## Data Availability

The data that support the findings of this study will be available from the authors upon reasonable request and with permission of Dr. Victor Dourado.

## Abbreviations

app: application
FVC: forced vital capacity
FEV1: forced expiratory volume in the first second
SMART: Sequential Multiple Assignment Randomized Trial
SPIRIT: Standard Protocol Items: Recommendations for Interventional Trials
